# Exercise Heart Rate During Training and Competitive Matches in Elite Soccer: More Questions than Answers

**DOI:** 10.3390/sports13120441

**Published:** 2025-12-08

**Authors:** Iwen Diouron, Cédric Leduc, Guilhem Escudier, Stéphane Perrey

**Affiliations:** 1 Montpellier Hérault Sports Club, 34000 Montpellier, France; iwen.diouron@umontpellier.fr (I.D.);; 2 EuroMov Digital Health in Motion, University Montpellier, IMT Mines Alès, 34090 Montpellier, France; 3 Centre for Human Performance, Carnegie School of Sport, Leeds Beckett University, Leeds LS2 3AX, UK

**Keywords:** cardiac, internal load, fitness, team sport, intensity, microcycle

## Abstract

Monitoring the training load of elite soccer players is a common practice for clubs. However, limited information exists about the internal load experienced by elite soccer players. The heart rate (HR) exposure of 51 French elite soccer players was monitored using conductive vests incorporating ECG bands during two consecutive seasons using a three-zone intensity model. HR exposure was broken down into volume (i.e., total time in the three zones) and intensity (i.e., relative time in the three zones). The effect of playing position, as well as the period (monthly or daily), was assessed. Regarding seasonal exposure, a significant difference was observed between key periods of the season (i.e., preseason, in season, end-of-season) for both volume and intensity (*p* < 0.05). Noteworthily, monthly HR exposure was relatively constant across competitive period. For weekly exposure, a significant difference in HR volume and intensity was observed between matches and training sessions (*p* < 0.001) potentially highlighting gaps in players’ readiness. Note that there were small variations in terms of HR exposure between the three first training days (*p* < 0.05), especially for time and relative time over 90% of maximal HR (not significant). This study not only provides insight into typical HR exposure in elite football but also questions the current training periodisation.

## 1. Introduction

Monitoring the dose or training load of elite soccer players is critical for clubs in terms of performance, health, and economics. Currently, the dose is broken down into two subdimensions: external (i.e., locomotor demand) and internal dose (i.e., psychophysiological responses) [1]. While external load monitoring has evolved at a fast pace due to the widespread use of wearable technologies, internal load, especially heart rate (HR)-related variables, has been overlooked [2,3].

This discrepancy may be due to a variety of reasons, ranging from theoretical and technological factors (e.g., signal analysis, wearability) [4,5] to logistical limitations inherent to elite sporting environments (e.g., schedule, poor buy-in from players and staff) [6,7]. Nevertheless, relying solely on external load measurement has limitations, as two similar external load exposures could lead to completely different HR responses due to high interindividual differences [3]. This could trigger different physiological adaptations [8]. Therefore, a better understanding of the typical internal load exposure (i.e., daily, weekly, and monthly) faced by elite soccer players monitored with HR would provide additional insight into current elite soccer periodization strategies.

Previous studies have reported an average HR value around 85% of its maximal (HR_max_) during a whole soccer match, with variations ranging from below 60% to near HR_max_ [9,10,11]. However, this information remains global and does not highlight the potential variability of HR between playing positions during matches, which is important for training prescriptions. Furthermore, an increase in match intensity has been observed since the beginning of the century [12], yet no recent studies have examined these trends regarding HR in the context of elite soccer.

Additionally, HR has been used to quantify internal dose during training sessions [13,14,15,16]. These studies revealed strong relationships between HR-based measures of dose (e.g., average HR value, TRIMP scores) and other internal training load indicators, such as session-rate of perceived exertion. Furthermore, HR-based measures are related to multiple external load markers, such as accelerations and high-intensity bursts [17]. However, these studies remain isolated and investigate HR-based measures on limited periods of time, which do not permit practitioners to have insights into the global exposure imposed on soccer players. Some studies also highlighted partial information regarding HR exposure (e.g., only time over 90% of HR_max_) [18,19]. Furthermore, the typical variables (e.g., Banister training impulse [20]) used to describe HR exposure could be questioned in the context of intermittent exercise such as soccer [21,22,23]. Consequently, there is still limited information regarding the typical HR exposure of elite soccer players during matches and training sessions. Therefore, the objectives of this study were to (1) describe the seasonal HR exposure of elite soccer players and how it evolves over the course of a competitive season; (2) highlight the typical HR exposure experienced by elite soccer players during a soccer microcycle, including matches. For both objectives, the effect of playing position will be assessed.

## 2. Materials and Methods

### 2.1. Study Design

**Figure 1:**
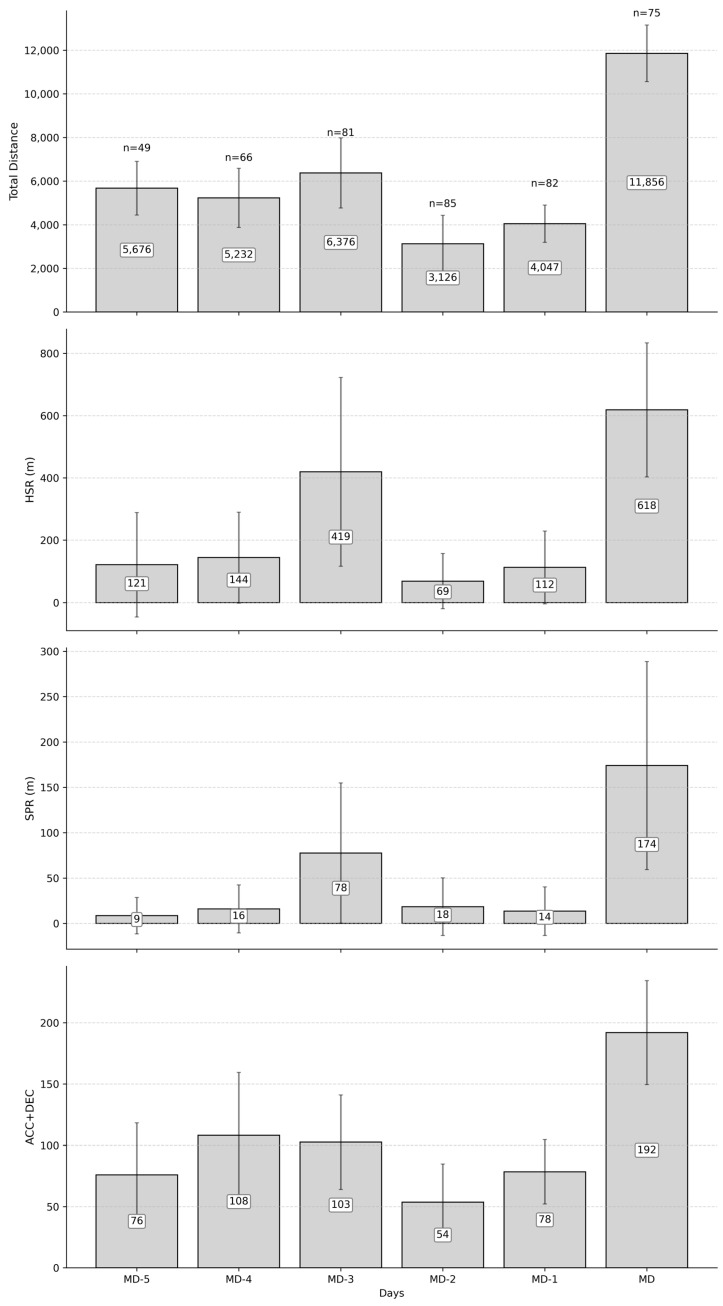
External load metrics faced by elite soccer players per day during the typical soccer microcycle of 2 consecutive seasons. This typical microcycle follows the tactical periodization principles, with the most observed type of training for MD-5, MD-4, MD-3, MD-1, and MD corresponding to aerobic (*n* = 37), strength (*n* = 38), endurance (*n* = 41), activation (*n* = 69), and match (*n* = 75) [24]. Only MD-2 varies across the season with different types of training (i.e., technical/tactical, speed, recovery). HSR, SPR, ACC + DEC stand for the distance covered between 19.8 and 25.2 km∙h^−1^, above 25.2 km∙h^−1^, and the sum of accelerations above 3 m∙s^−2^, and decelerations below 3 m∙s^−2^, respectively.

The study conducted for this research article took place over two soccer competitive seasons (i.e., 2023–2024 and 2024–2025) of an elite French soccer team competing at the highest national level (i.e., Ligue 1). During each season, the studied team underwent a 6-week preseason period at the beginning of the season consisting of physiological assessment, physical conditioning, competition preparation, and friendly matches. This was followed by two competitive periods from August to December and from January to May, with a week without training interspersed between them. During a major part of the competitive periods (i.e., excluding congested periods and international breaks), the weekly training programme followed a typical tactical periodization structure described in a press article [24]. Specifically, these weeks contained a match, a rest day, and five training sessions with a specific focus (Figure 1). The five training sessions were oriented towards aerobic (i.e., low to medium intensity training with technical drills, possessions), strength (i.e., intensive sessions with rondos and small-sided games), endurance (i.e., extensive sessions with tactical drills, large-sided games), tactical or speed and activation (i.e., short sessions with tactical drills and small-sided games) for MD-5, MD-4, MD-3, MD-2, and MD-1, respectively. During the studied period, internal dose has been monitored through exercise HR.

### 2.2. Participants

Fifty-one French elite professional soccer players competing at the highest level (i.e., Ligue 1) initially took part in this study and were categorized according to their playing position (i.e., central defenders [CD], wide defenders [WD], central midfielders [CM], and forwards [FW]) (Table 1). Twenty-three of the studied players were involved in the study over two consecutive seasons. Goalkeepers were excluded from the study. As they play at the national or international level, the studied players were considered between highly trained and elite athletes [25]. As the data collected arose from the players’ daily monitoring, which is required for their professional practice, ethical committee approval was not necessary [26]. The players’ data were anonymized, and the study complied with the recommendations of the Declaration of Helsinki.

### 2.3. Data Collection

The internal dose of each player was measured using a valid HR sensor sampled at 1 Hz (Polar H10, Polar, Kempele, Finland) [27] placed in a specially designed vest (Statsports conductive vest, Statsports, Newry, Northern Ireland) connected to a recorder (i.e., global positioning system [GPS] unit) through Bluetooth. Each player wore the same HR sensor for every session during both seasons to avoid inter-unit variability. Only reliable signals were included in the study (i.e., corresponding to 96.0% of the full season dataset). The quality of the signal was manually checked by visualizing the HR data, and the signal was removed when an interruption or an erroneous HR signal (i.e., not following the expected trends) corresponding to a minimum of 25% of the session was observed. Therefore, all the sessions studied were included, as only single players’ sessions were excluded.

Due to the intermittent nature of soccer and the type of data available during the two seasons, it was decided to categorize exercise intensity into six arbitrary zones, each representing 10% of the individual ${HR}_{max}$ (Table 2) [28]. ${HR}_{max}$ was defined based on the maximal value observed during an incremental exhaustion test performed at the beginning of each season and was regularly updated regarding new maximal values observed during training sessions and matches. On the one hand, HR volume was defined as the total time spent in zones. On the other hand, previous HR monitoring strategies used the average HR value over a defined period, with the higher value the higher intensity and conversely [20]. However, the stochastic nature of soccer potentially creates high short HR peaks, followed by low HR values might produce misleading analysis with the use of average HR. Therefore, when quantifying the internal intensity using HR, the percentage of session time spent in a specific intensity zone (i.e., relative time) might represent an informative measure of intensity, with a higher relative time spent in the high-intensity zone reflecting greater intensity, and conversely. To ensure a higher practical and physiological relevance, 3 zones delimitating low (LI), moderate (MI), and high (HI) intensity were defined, with zones 1 to 4, 5, and 6 of Edwards’ zones corresponding to LI, MI, and HI, respectively (Table 2). This is because 80 and 90% are the nearest values to ventilatory thresholds, highlighting a physiological difference in exercise intensity (Ref. [29], *unpublished observations*).

### 2.4. Statistical Analyses

Statistical analyses were conducted using Python software (3.11.7) (Python, Wilmington, DE, USA). Typical training and match exposures were primarily characterized by the average time spent in intensity zones per player, along with the associated standard deviation. Linear mixed models were used to assess the effect of period and playing position on HR exposure. The chosen period (i.e., month, day) and playing position were defined as fixed effects, while the player identification code was defined as the random effect to account for the repeated measurements. A minimum of 10 sessions per month was arbitrarily set to be considered in the monthly analysis to ensure enough data and avoid long absences (e.g., injuries). Additionally, only team training was considered (i.e., no rehabilitation or individual training). Player position was converted into a numerical code to assess the overall effect of player position and was centred to suppress the random order of positions and ensure a better analysis of the intercept [30]. Statistical significance was set at *p* < 0.05. When a significant effect was found, a post hoc analysis using a linear mixed model allowing for a pairwise comparison was conducted, and the effect size was calculated using an adjusted Cohen’s d, based on dividing the value of the fixed effects coefficient by the square root of the sum of the residual and random variance [31]. The magnitude of effect size was defined as <0.01 (trivial); 0.01–0.20 (very small); 0.20–0.49 (small); 0.50–0.79 (moderate); 0.80–1.19 (large); 1.20–1.99 (very large); and >2.0 (extremely large) [32].

## 3. Results

### 3.1. Monthly HR Exposure

#### 3.1.1. Volume

Table 3 presents the typical HR exposure in terms of volume for the two competitive seasons. First, a significant effect of playing position was observed for LI (*p* < 0.01) (Supplementary Figure S1) with FW spending more time at LI compared to CD (*p* < 0.01; moderate) and WD (*p* < 0.05; small); and CM spending more time at LI in comparison to WD (*p* < 0.05; small) and CD (*p* < 0.001; moderate).

**Figure 2:**
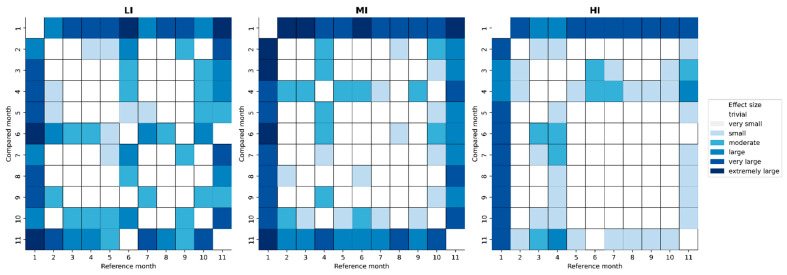
Heatmap of the pairwise comparison between months for the training volume measured through heart rate (HR) during two consecutive seasons of an elite soccer team. Coloured cells correspond to significant differences (*p* < 0.05), and the colour highlights the value of the effect size (i.e., adjusted Cohen’s d). LI, MI, and HI stand for the time spent below 80%, between 80% and 90%, and above 90% of maximal HR, respectively. Trivial, very small, small, moderate, large, very large, and extremely large correspond to an adjusted Cohen’s d value of <0.01, 0.01–0.2, 0.2–0.5, 0.5–0.8, 0.8–1.2, and >1.2, respectively.

Then, a significant effect of month (M) on volume was observed for all zones (*p* < 0.01). The HR volume during M1 is a major component of this negative effect, as it was shown to be significantly higher in all zones compared to the other months (*p* < 0.001; large to extremely large). On the contrary, the HR volume during M11 is lower than during any other month in every zone (*p* < 0.05; small to extremely large) except during M6 for LI and HI. Figure 2 summarizes pairwise comparisons between months and their associated effect size when significant.

#### 3.1.2. Intensity

**Figure 3:**
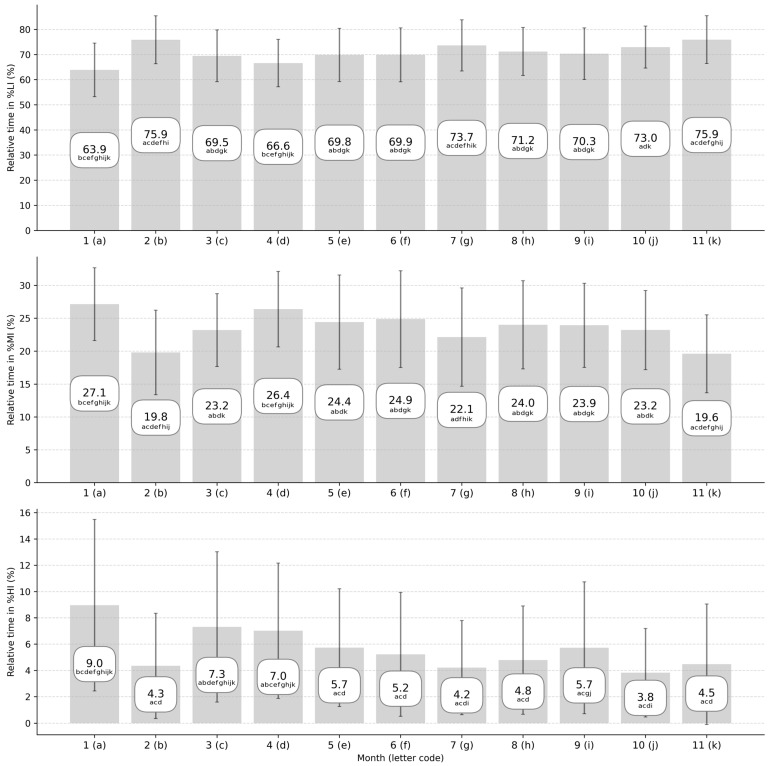
Repartition of relative training time spent in heart rate (HR) zone (in percentage of total training time) averaged per month during two consecutive elite soccer seasons. The standard deviation of each month is described by error bars. %LI, %MI, and %HI represent the percentage of training time spent between 0% and 80%, 80% and 90%, and 90% and 100% of players’ maximal HR. a, b, c, d, e, f, g, h, i, j, and k correspond to a significant difference with months 1, 2, 3, 4, 5, 6, 7, 9, 10, and 11, respectively.

Figure 3 summarizes the relative time spent in HR zones per month across the two competitive seasons and the significant differences observed between months (*p* < 0.05, small to very large). The associated effect sizes of interday differences are described in Supplementary Figure S2. No significant difference was observed for playing position.

### 3.2. Weekly HR Exposure

#### 3.2.1. Volume

**Figure 4:**
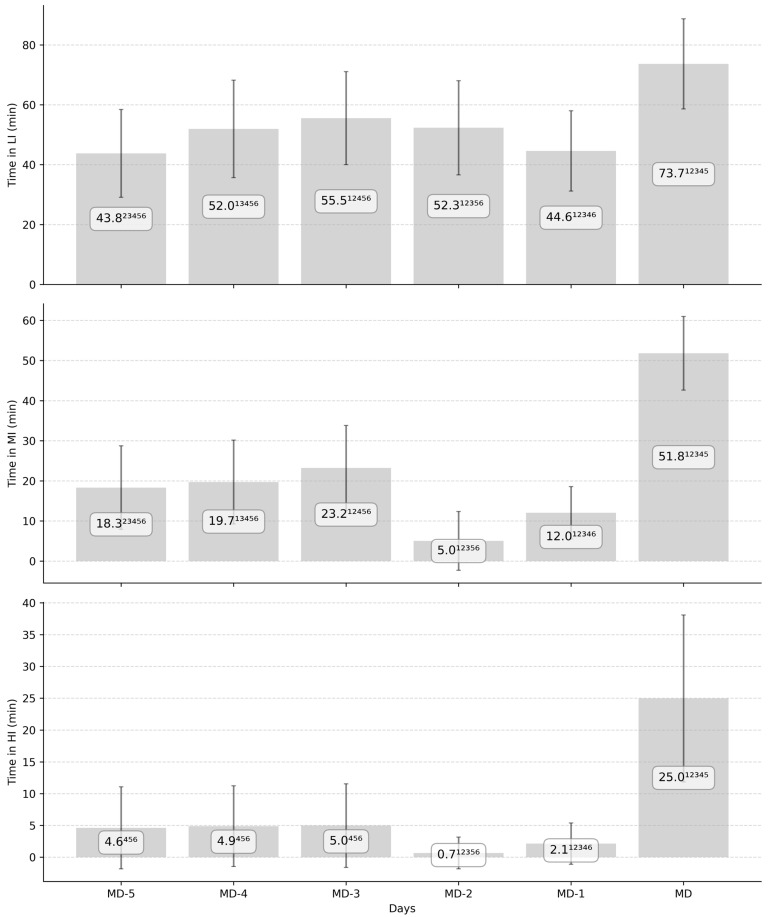
Absolute time spent in heart rate (HR) zones regarding the number of days before the next match. The presented results highlight the average time spent in each HR zone and the associated standard deviation measured in an elite soccer team following a tactical periodization during two consecutive seasons. MD-5, MD-4, MD-3, MD-2, and MD-1 correspond to the training day with 5, 4, 3, 2, 1 days until the next match, while MD corresponds to the match day. LI, MI, and HI express the time spent between 0% and 80%, 80% and 90%, and 90% and 100% of maximal players’ HR. Numbers 1, 2, 3, 4, 5, and 6 describe a significant difference with MD-5, MD-4, MD-3, MD-2, MD-1, and MD, respectively (*p* < 0.001; small to extremely large).

Figure 4 describes the average time spent in the different HR zones over the course of a typical training week in elite soccer per day (MD) before the match and per playing position. A significant effect of the position was observed for MI with CD showing a higher value compared to FW (*p* < 0.01; small) and for HI with CD showing a higher value compared to FW and CM (*p* < 0.05; small). A significant effect of day was observed for all zones (*p* < 0.001) (Figure 2). The associated effect sizes of interday differences are described in Supplementary Figure S3.

Additionally, the difference between cumulated training sessions and match HR exposure was explored. The results are shown in Table 4.

#### 3.2.2. Intensity

**Figure 5:**
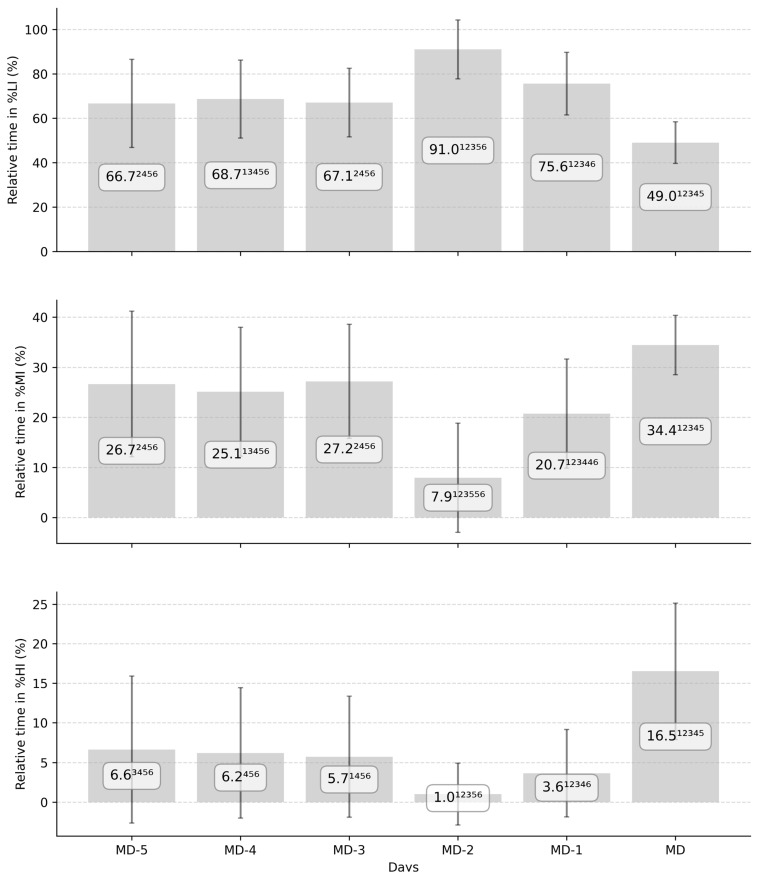
Repartition of average relative training time spent in heart rate (HR) zone (in percentage of total time) per days before the match and the associated standard deviation during two consecutive elite soccer seasons. MD-5, MD-4, MD-3, MD-2, and MD-1 correspond to the training day with 5, 4, 3, 2, and 1 days until the next match, while MD corresponds to the match day. %LI, %MI, and %HI represent the percentage of training time spent between 0% and 80%, 80% and 90%, and 90% and 100% of players’ maximal HR. Numbers 1, 2, 3, 4, 5, 6 describe a significant difference with MD-5, MD-4, MD-3, MD-2, MD-1, and MD, respectively (*p* < 0.01; very small to extremely large).

Figure 5 highlights the repartition of the total training session time in HR zones during a typical elite soccer microcycle. A significant effect of playing position was observed for all the zones (*p* < 0.05), with CD showing lower %LI compared to FW and CM, while describing higher %MI, %HI compared to FW and CM, respectively (*p* < 0.05; small). A significant effect of day on all the zones was observed (*p* < 0.01) and described in Figure 3. The associated effect sizes of interday differences are described in Supplementary Figure S4.

### 3.3. Match HR Exposure

Table 5 summarizes the average match HR exposure (i.e., for 90 min) per playing position faced by elite soccer players in the national championship during the two studied seasons (*n* = 67 matches). No significant effect of field position was observed for all zones.

## 4. Discussions

The objectives of this study were to (1) describe the monthly HR exposure of elite soccer players and how it evolves over the course of a competitive season; (2) highlight the typical HR exposure experienced by elite soccer players during a soccer microcycle, including matches. The main findings of this study were (1) a significant difference HR exposure between key periods of the elite soccer season for both volume and intensity, and (2) a significant difference in HR exposure between matches and training sessions.

### 4.1. Monthly HR Exposure

This study revealed a significant difference in HR exposure (i.e., volume and intensity) between preseason, competitive period, and end-of-season. First, the difference in HR volume could be due to a difference in total training time. As preseason is a period where physical improvement is central [33], it was logical to observe a significant increase in total training time (Supplementary Figure S5). Furthermore, the end of the championship led to a decrease in total training time during M11. Similarly, M6 total training time decreased similarly to M11 due to a winter break (i.e., one week). These might be due to the widely observed multicollinearity in training load indicators [34]. Therefore, if practitioners want to accumulate high HR volume, they can consider manipulating total training time.

While the increased intensity (i.e., higher relative time in HI and MI and lower relative time in LI) during M1 could be explained by the emphasis on physical development during preseason [33], players’ physical status might be another factor [35,36]. Indeed, the off-season (i.e., period without soccer training sessions) was between 4 and 6 weeks during this study and was associated with a short-term period of inactivity (~2 weeks). Such a timeframe has been shown to be associated with a decrease in aerobic fitness (e.g., reduced blood and stroke volume [36]), explaining the increased HR [37,38]. In contrast, M2 showed a significantly lower intensity compared to the other months. The tapering strategy (i.e., reduced load) used ahead of the beginning of the competitive season might explain the current results [39]. It may also reveal an increase in aerobic fitness (e.g., increased running economy, increased parasympathetic activity, cardiac alterations), which decreases HR [5,40]. However, the exact causal links have not been investigated and require further studies.

Even if significant differences for both HR volume and intensity were observed between the months of the competitive period, it seems that the effect sizes were generally trivial to moderate compared with M1 or M11. This lack of monthly variation was observed for external load (e.g., total distance, distance over 5.5 m∙s^−1^) in another elite soccer team [41]. The weekly fixture (i.e., 1 or 2 matches every week) forces practitioners to shift training content towards the technical component and tactical preparation while maintaining physical qualities [33]. Additionally, the total training time was maintained during the competitive season (Supplementary Figure S5), confirming the previous suggestion about the influence of training time on HR exposure over the preseason.

Overall, while the evolution of HR exposure at the end of the preseason appeared to be consistent with a hypothetical change in fitness, its maintenance during the competitive period remains elusive. This questions the effect of such a periodization strategy and/or weekly fixture organization on players’ fitness. Therefore, there is a need to assess players’ fitness on a regular basis to monitor the effectiveness of periodization strategies at the team and individual levels.

### 4.2. Weekly HR Exposure

This study described a significant difference in HR exposure on match day compared to all other training days, with a significantly higher volume and intensity. Even after cumulating training sessions, HR volume (i.e., average of 17.3 min) did not reach match demands (i.e., average of 25.0 min) for HI (Table 4). The current results are lower than a previous study conducted on Dutch professional players during the 2014–2015 season. It had shown a ratio greater than 1 between cumulated training sessions (i.e., average of 28 min) and match exposure (i.e., average of 22 min) in HI [19]. In contrast, the studied team reported ratios greater than 1.4 for commonly used external load metrics (e.g., distance over 20 km∙h^−1^, number of accelerations) (unpublished observations). These values are regularly observed in elite soccer [42]. Therefore, the current results highlight for the first time important gaps between training sessions and match HR exposure in elite soccer. While the current study was conducted in a unique environment, it might highlight a possible change in soccer training strategies whereby internal load is slightly overlooked in the weekly planning process [2,3,38]. As it was outlined in the previous section, the total session time could also explain this difference between training and match HR exposure (e.g., an average of 150 min for a match session [including warm-up] compared to an average of 90 min for MD-3, which is the longest training session). Overall, the observed gap might question the readiness of players regarding matches’ physiological demands.

Significant differences in volume and intensity were shown between MD-1 and MD-2 compared to MD-3, MD-4, and MD-5, revealing similar results to previous studies [43,44]. However, while some significant differences were observed between D-5 (i.e., aerobic), D-4 (i.e., strength), and D-3 (i.e., endurance) for LI and MI volume, no significant difference for HI was reported. Additionally, intensity analysis did not reveal significant differences for these days or without a relevant effect size. Yet, a preceding study highlighted variation in time in HI during the week, with a peak occurring at D-4 [19]. Similarly, a variation in external load has been reported with a peak observed in the middle of the week [41,43,44,45]. The current results might confirm our hypothesis on the neglect of HR exposure within training strategy over the past decade. However, while the external load alternation was linked with different acute neuromuscular responses [18], such experiments have not been conducted on cardiovascular responses. Therefore, while the lack of variation between training sessions could be intriguing from the players’ conditioning perspective, it might also question the specificity of current HR variables to monitor internal load in soccer (e.g., alteration in relationship with other internal markers [21], high variation in HR due to the stochastic nature of soccer). Such questions need to be investigated by the concomitant analysis of HR exposure and physiological adaptations at different time scales (i.e., acute and chronic training effects).

### 4.3. Match HR Exposure

The analysis of match exposure did not highlight significant differences between playing positions regarding time spent in HR zones. Though, previous studies highlighted significant differences between positions for HR exposure during competitive matches [46] and during maximal intensity periods [47]. However, these studies were conducted on youth players. Therefore, maturation status might need to be considered when comparing their results to the current study [48]. Additionally, significant differences regarding external load between positions have been widely observed in soccer [49,50,51], suggesting the need for a position-based approach to training [52,53]. In regards of HR training exposure, the results of the present study are relatively paradoxical. Indeed, clear variations have been shown regarding training, while match exposure was similar between playing positions. While the recent hyper focus on external load variables might offer some insights into neuromuscular load [2], it can question the readiness of certain players from a cardiovascular standpoint, and to an extent, internal load periodization strategy.

### 4.4. Current Limitations and Perspectives

Despite the new findings of this study, several limitations need to be mentioned. First, the use of arbitrary zones does not highlight the individual physiological specificity [54]. Some dose monitoring strategies using individualized intensity zones based on ventilatory or lactate thresholds might improve HR exposure characterization in elite soccer [23,29]. Second, no other indicators of internal load were used and compared to HR exposure. Indeed, while HR relationship with exercise intensity during continuous exercise is strong, it might be altered during intermittent exercise, such as soccer [21], and make the analysis of HR complex. The use of blood markers, such as blood lactate during soccer, might offer some perspectives on intensity characterization. Furthermore, measures of players’ response to the applied dose (e.g., maximal or submaximal running test) need to be considered to answer the questions and confirm the gaps currently exposed in this article. Finally, despite using a large cohort of players (*n* = 51) over a long period (i.e., two seasons), this study focused on a single team within a specific club, which might not reflect the whole elite soccer player population. Therefore, future studies analyzing HR exposure in other contexts are needed to confirm these preliminary observations.

## 5. Conclusions and Practical Applications

This study aimed to report the current monthly, weekly, and match HR exposure of elite soccer players. First, HR exposure analysis revealed different insights into training load compared to common observations made through the sole scope of external load. Therefore, it is recommended to consider internal load concurrently with external load. It will permit practitioners to increase their scope of information about players’ training load and individual specificity, thus potentially optimizing players’ conditioning.

Second, the current results have highlighted important gaps between matches and training sessions in HR exposure. Similarly, there exists a certain monotony in training content regarding HR exposure at different time scales (e.g., over months during the competitive period, between training days). This potentially questions the capacity of current soccer training content to trigger the required physiological adaptations. While these observations might be due to the neglect of internal load indicators in the weekly training periodization over the past years, they could reveal potential ways to improve players’ conditioning. Designing and implementing a training strategy with the objective of reducing this discrepancy and monotony (e.g., longer training and/or higher training intensities) could lead to an optimized players’ readiness. However, there is a need to monitor players’ physiological adaptations regularly to confirm this hypothesis.

While this study gives several propositions to increase elite soccer players’ conditioning using HR exposure, a deeper analysis of typical soccer training drills HR exposure (e.g., small-, medium- or large-sided games) might be interesting to pursue, as it could give a kind of toolbox to practitioners for designing training sessions.

## Figures and Tables

**Table 1:** Description of the studied population per season.

Variables		Season 1 (2023–2024)	Season 2 (2024–2025)
N	Overall	37	37
	CD	8	9
	WD	7	6
	CM	9	11
	FW	13	11
Age (years)	Overall	24.0 ± 4.4	24.5 ± 5.1
	CD	24.8 ± 5.3	24.6 ± 5.3
	WD	24.3 ± 4.3	24.9 ± 4.7
	CM	23.8 ± 4.8	24.4 ± 5.6
	FW	23.4 ± 4.1	24.3 ± 5.5
Height (cm)	Overall	181.8 ± 6.6	181.0 ± 6.5
	CD	189.1 ± 4.5	188.3 ± 5.0
	WD	180.3 ± 2.6	178.2 ± 3.8
	CM	179.3 ± 6.8	178.6 ± 5.3
	FW	179.7 ± 6.3	178.8 ± 5.6
Body mass (kg)	Overall	77.0 ± 7.4	77.3 ± 7.2
	CD	82.7 ± 7.3	83.2 ± 5.3
	WD	74.0 ± 6.4	70.9 ± 3.9
	CM	74.5 ± 5.8	74.3 ± 3.3
	FW	77.0 ± 7.7	78.9 ± 8.7
Maximal HR (bpm)	Overall	194.2 ± 9.1	196.1 ± 7.5
	CD	186 ± 6.3	192.4 ± 9.2
	WD	194.7 ± 10.2	197.7 ± 6.9
	CM	198.2 ± 8.1	197.5 ± 6.3
	FW	196.2 ± 8.3	196.9 ± 7.5

Abbreviations: HR stands for heart rate; CD, WD, CM, and FW stand for central defenders, wide defenders, central midfielders, and forwards, respectively.

**Table 2:** Heart rate intensity zones, adapted from [28].

Modified Intensity Zone	Edwards’ Intensity Zone	Percentage of Maximal Heart Rate
Low intensity	1	0–40%
	2	40–60%
	3	60–70%
	4	70–80%
Moderate intensity	5	80–90%
High intensity	6	90–100%

**Table 3:** Description of the time spent in heart rate (HR) zones by an elite soccer team per month during two consecutive seasons. Data are presented in minutes as average, standard deviation, and range. Low, moderate, and high intensity correspond to time spent <80% of maximal HR, between 80% and 90% of maximal HR, and >90% of maximal HR, respectively.

Months	Low Intensity				Moderate Intensity				High Intensity			
	CD	WD	CM	FW	CD	WD	CM	FW	CD	WD	CM	FW
1 **	1112 ± 417 [362, 1840]	1077 ± 388 [510, 1667]	1034 ± 379 [514, 1722]	1232 ± 507 [362, 1840]	549 ± 136 [318, 710]	503 ± 166 [167, 763]	439 ± 144 [190, 725]	405 ± 118 [156, 619]	178 ± 89 [75, 294]	187 ± 129 [46, 372]	130 ± 80 [39, 299]	106 ± 86 [17, 284]
2 *	833 ± 122 [632, 1030]	785 ± 139 [548, 956]	907 ± 164 [516, 1154]	946 ± 153 [715, 1200]	274 ± 66 [181, 363]	269 ± 90 [128, 374]	227 ± 89 [78, 368]	190 ± 84 [40, 323]	54 ± 31 [10, 93]	88 ± 60 [32, 203]	45 ± 58 [0, 223]	36 ± 42 [0, 126]
3	661 ± 247 [342, 968]	607 ± 126 [450, 812]	946 ± 170 [679, 1209]	834 ± 265 [373, 1185]	242 ± 91 [117, 394]	283 ± 106 [155, 401]	267 ± 62 [180, 345]	249 ± 78 [183, 411]	81 ± 61 [2, 177]	108 ± 64 [21, 185]	65 ± 67 [4, 241]	80 ± 69 [5, 238]
4	626 ± 66 [543, 712]	696 ± 135 [419, 895]	862 ± 216 [448, 1155]	869 ± 140 [612, 1041]	255 ± 92 [104, 327]	356 ± 144 [181, 554]	313 ± 84 [227, 529]	315 ± 94 [159, 483]	93 ± 64 [3, 166]	11 ± 75 [21, 279]	66 ± 60 [8, 228]	78 ± 83 [8, 280]
5	664 ± 151 [505, 837]	679 ± 182 [393, 990]	852 ± 194 [414, 1137]	839 ± 181 [514, 1152]	270 ± 140 [98, 486]	305 ± 118 [91, 428]	258 ± 94 [127, 445]	275 ± 82 [164, 409]	103 ± 54 [21, 164]	82 ± 53 [5, 154]	38 ± 27 [5, 82]	63 ± 67 [9, 217]
6	553 ± 126 [407, 827]	586 ± 111 [438, 772]	781 ± 162 [584, 1072]	706 ± 128 [407, 827]	256 ± 75 [129, 350]	227 ± 94 [108, 384]	215 ± 66 [123, 323]	256 ± 105 [128, 489]	80 ± 60 [5, 179]	54 ± 31 [8, 86]	24 ± 23 [2, 79]	43 ± 37 [1, 104]
7	737 ± 217 [406, 1100]	834 ± 215 [524, 1117]	1060 ± 151 [785, 1318]	797 ± 170 [477, 1070]	314 ± 102 [145, 500]	203 ± 103 [83, 333]	282 ± 116 [104, 476]	239 ± 103 [76, 477]	80 ± 59 [38, 231]	22 ± 16 [8, 39]	38 ± 37 [4, 138]	53 ± 56 [1, 182]
8	620 ± 33 [579, 655]	822 ± 106 [648, 1028]	956 ± 99 [772, 1083]	830 ± 117 [685, 1012]	319 ± 95 [173, 404]	325 ± 100 [160, 452]	261 ± 74 [177, 387]	260 ± 91 [171, 410]	79 ± 87 [15, 225]	75 ± 50 [8, 142]	45 ± 49 [3, 170]	46 ± 36 [9, 123]
9	645 ± 202 [438, 990]	649 ± 142 [487, 810]	876 ± 151 [588, 1110]	799 ± 164 [456, 984]	277 ± 119 [81, 442]	282 ± 115 [155, 458]	269 ± 83 [151, 420]	229 ± 98 [121, 427]	90 ± 68 [0, 173]	82 ± 49 [27, 164]	50 ± 53 [9, 199]	36 ± 28 [8, 83]
10	833 ± 187 [639, 1180]	887 ± 154 [569, 1059]	1010 ± 216 [686, 1325]	905 ± 199 [505, 1157]	344 ± 67 [279, 480]	326 ± 79 [196, 409]	278 ± 85 [143, 438]	235 ± 84 [134, 401]	62 ± 60 [15, 196]	82 ± 52 [18, 174]	36 ± 41 [0, 130]	25 ± 18 [8, 58]
11	559 ± 94 [459, 676]	561 ± 113 [446, 774]	673 ± 105 [435, 782]	607 ± 92 [501, 669]	170 ± 21 [149, 196]	175 ± 57 [74, 227]	140 ± 47 [59, 201]	138 ± 28 [106, 155]	37 ± 26 [15, 70]	57 ± 57 [1, 167]	25 ± 25 [0, 77]	15 ± 3 [12, 17]

** denotes that all the month was part of the preseason; * denotes that only the first 2 weeks of the month were part of the preseason. Abbreviations: CD for central defenders; WD for wide defenders; CM for central midfielders; and FW for forwards.

**Table 4:** Cumulative average training session heart rate (HR) exposure during the typical microcycle compared to average HR exposure during match sessions.

Zone	Match (min)	Training (min)	Difference (min)	Ratio
**Low intensity**	73.7	248.2	174.5	3.4
**Moderate intensity**	51.8	78.3	26.5	1.5
**High intensity**	25.0	17.3	−7.7	0.7

Low, moderate, and high intensity correspond to time spent <80% of maximal HR, between 80 and 90% of maximal HR, and >90% of maximal HR, respectively.

**Table 5:** Description of full match heart rate (HR) exposure in elite soccer players measured over two consecutive seasons. All the matches included correspond to the national championship (*n* = 67). The data are presented as the average, standard deviation, and range. The game was considered full when the match total time was over 90 min. Low, moderate, and high intensity correspond to time spent <80% of maximal HR, between 80% and 90% of maximal HR, and >90% of maximal HR, respectively.

Field Position	Low Intensity		Moderate Intensity		High Intensity	
	Volume (min)	Intensity (%)	Volume (min)	Intensity (%)	Volume (min)	Intensity (%)
**CD**	30.2 ± 10.0 [6.65, 63.4]	30.4 ± 9.9 [6.9, 63.9]	46.2 ± 7.5 [28.4, 62.8]	46.7 ± 7.4 [27.7, 65.7]	22.1 ± 12.3 [0.0, 60.0]	22.4 ± 12.5 [0.0, 62.4]
**WD**	25.4 ± 9.2 [3.5, 51.5]	25.8 ± 9.4 [3.6, 52.5]	44.8 ± 6.7 [21.6, 65.7]	45.5 ± 7.0 [22.4, 67.7]	28.3 ± 11.6 [5.6, 71.0]	28.6 ± 11.7 [5.7, 73.9]
**CM**	22.5 ± 8.8 [8.2, 45.2]	22.9 ± 9.0 [8.4, 47.0]	47.4 ± 7.1 [28.7, 66.0]	48.3 ± 7.2 [28.8, 66.1]	28.2 ± 12.5 [6.3, 56.4]	28.7 ± 12.7 [6.7, 56.6]
**FW**	33.0 ± 10.6 [13.7, 54.2]	33.6 ± 10.6 [14.3, 55.3]	44.4 ± 7.8 [31.5, 60.5]	45.3 ± 8.1 [30.1, 63.1]	20.5 ± 9.3 [2.9, 41.4]	20.9 ± 9.5 [3.1, 42.4]
**Overall**	27.3 ± 10.3 [3.5, 36.4]	27.6 ± 10.3 [3.6, 63.9]	45.9 ± 7.3 [21.6, 66.0]	46.7 ± 7.4 [22.4, 67.7]	25.1 ± 12.2 [0.0, 71.0]	25.5 ± 12.4 [0.0, 73.9]

Abbreviations: CD for central defenders (*n* = 121); WD for wide defenders (*n* = 92); CM for central midfielders (*n* = 107); and FW for forwards (*n* = 57).

## Data Availability

The data presented in this study are available on request from the corresponding author due to ethical restrictions imposed by the institutional review board to protect participant confidentiality.

## Supplementary Materials

The following supporting information can be downloaded at: https://www.mdpi.com/article/10.3390/sports13120441/s1. Supplementary Figure S1: Evolution of monthly HR exposure per playing position during two consecutive seasons of an elite soccer team. Lines and areas represent the average time and 95% confidence interval. CD, WD, CM, and FW correspond to central defenders, wide defenders, central midfielders, and forwards. LI, MI, and HI correspond to low, moderate, and high intensity zones, respectively; Supplementary Figure S2. Heatmap of the pairwise comparison between months for training intensity measured through heart rate (HR) during two consecutive seasons of an elite soccer team. Coloured cells correspond to significant differences (*p* < 0.05) and the colour highlights the value of the effect size (i.e., adjusted Cohen’s d). LI_perc, MI_perc, and HI_perc stand for the relative time spent below 80%, between 80% and 90% and above 90% of maximal HR, respectively. Trivial, very small, small, moderate, large, very large, and extremely large correspond to an adjusted Cohen’s d value of <0.01, 0.01–0.2, 0.2–0.5, 0.5–0.8, 0.8–1.2, and >1.2, respectively; Supplementary Figure S3. Heatmap of the pairwise comparison between days for training volume measured through heart rate (HR) during the typical microcycle of an elite soccer team. The data presented is from two consecutive soccer seasons. Coloured cells correspond to significant differences (*p* < 0.05) and the colour highlights the value of the effect size (i.e., adjusted Cohen’s d). MD-5, MD-4, MD-3, MD-2, and MD-1 correspond to the training day with 5, 4, 3, 2, and 1 days until the next match, while MD corresponds to the match day. LI, MI, and HI stand for the time spent below 80%, between 80% and 90%, and above 90% of maximal HR, respectively. Trivial, very small, small, moderate, large, very large, and extremely large correspond to an adjusted Cohen’s d value of <0.01, 0.01–0.2, 0.2–0.5, 0.5–0.8, 0.8–1.2, and >1.2, respectively; Supplementary Figure S4. Heatmap of the pairwise comparison between days for training intensity measured through heart rate (HR) during the typical microcycle of an elite soccer team. The data presented is from two consecutive soccer seasons. Coloured cells correspond to significant differences (*p* < 0.05) and the colour highlights the value of the effect size (i.e., adjusted Cohen’s d). MD-5, MD-4, MD-3, MD-2, and MD-1 correspond to the training day with 5, 4, 3, 2, and 1 days until the next match, while MD corresponds to the match day. LI_perc, MI_perc, and HI_perc stand for the relative time spent below 80%, between 80% and 90%, and above 90% of maximal HR, respectively. Trivial, very small, small, moderate, large, very large, and extremely large correspond to an adjusted Cohen’s d value of <0.01, 0.01–0.2, 0.2–0.5, 0.5–0.8, 0.8–1.2, and >1.2, respectively; Supplementary Figure S5. Evolution of the monthly average total training time and the associated standard deviation during two consecutive elite soccer seasons (A) and the heatmap of the pairwise comparison between months (B). Coloured cells correspond to significant differences (*p* < 0.05) and the colour highlights the value of the effect size (i.e., adjusted Cohen’s d). Trivial, very small, small, moderate, large, very large, and extremely large correspond to an adjusted Cohen’s d value of <0.01, 0.01–0.2, 0.2–0.5, 0.5–0.8, 0.8–1.2, and >1.2, respectively.
