# Supplementary material for: Exercise Heart Rate During Training and Competitive Matches in Elite Soccer: More Questions than Answers
Source: Sports (Basel). 2025 Dec 8;13(12):441. doi: 10.3390/sports13120441 (PMC12737241; doi:10.3390/sports13120441)
Supplement: Supplementary file 1 [file sports-13-00441-s001.zip › Legend - Supplementary materials.pdf]

Supplementary materials

Supplementary Figure S1. Evolution of monthly HR exposure per playing position during 2 consecutive seasons of an elite soccer team. Lines and areas represent the average time and 95% confidence interval. CD, WD, CM and FW correspond to central defenders, wide defenders, central midfielders and forwards. LI, MI and HI correspond to low, moderate and high intensity zones, respectively.

Supplementary Figure S2. Heatmap of the pairwise comparison between months for training intensity measured through heart rate (HR) during 2 consecutive seasons of an elite soccer team. Coloured cells correspond to significant differences ($p<0.05$) and the colour highlight the value of the effect size (i.e., adjusted Cohen's d). LI_perc, MI_perc and HI_perc stand for the relative time spent below 80%, between 80% and 90% and above 90% of maximal HR, respectively. Trivial, very small, small, moderate, large, very large and extremely large corresponds to an adjusted Cohen's d value of <0.01, 0.01-0.2, 0.2-0.5, 0.5-0.8, 0.8-1.2 and >1.2, respectively.

Supplementary Figure S3. Heatmap of the pairwise comparison between day for training volume measured through heart rate (HR) during the typical microcyle of an elite soccer team. The data presented provides from 2 consecutive soccer seasons. Coloured cells correspond to significant differences ($p<0.05$) and the colour highlight the value of the effect size (i.e., adjusted Cohen's d). MD-5, MD-4, MD-3, MD-2, MD-1 corresponds to the training day with 5, 4, 3, 2, 1 days until the next match while MD corresponds to the match day. LI, MI and HI stand for the time spent below 80%, between 80% and 90% and above 90% of maximal HR, respectively. Trivial, very small, small, moderate, large, very large and extremely large corresponds to an adjusted Cohen's d value of <0.01, 0.01-0.2, 0.2-0.5, 0.5-0.8, 0.8-1.2 and >1.2, respectively.

Supplementary Figure S4. Heatmap of the pairwise comparison between day for training intensity measured through heart rate (HR) during the typical microcyle of an elite soccer team. The data presented provides from 2 consecutive soccer seasons. Coloured cells correspond to significant differences (p<0.05) and the colour highlight the value of the effect size (i.e., adjusted Cohen's d). MD-5, MD-4, MD-3, MD-2, MD-1 corresponds to the training day with 5, 4, 3, 2, 1 days until the next match while MD corresponds to the match day. LI_perc, MI_perc and HI_perc stand for the relative time spent below 80%, between 80% and 90% and above 90% of maximal HR, respectively. Trivial, very small, small, moderate, large, very large and extremely large corresponds to an adjusted Cohen's d value of <0.01, 0.01-0.2, 0.2-0.5, 0.5-0.8, 0.8-1.2 and >1.2, respectively.

Supplementary Figure S5. Evolution of monthly average total training time and the associated standard deviation during 2 consecutive elite soccer season (A) and the heatmap of the pairwise comparison between month (B). Coloured cells correspond to significant differences (p<0.05) and the colour highlight the value of the effect size (i.e., adjusted Cohen's d). Trivial, very small, small, moderate, large, very large and extremely large corresponds to an adjusted Cohen's d value of <0.01, 0.01-0.2, 0.2-0.5, 0.5-0.8, 0.8-1.2 and >1.2, respectively.
