# Supplementary figures and images for: Exercise Heart Rate During Training and Competitive Matches in Elite Soccer: More Questions than Answers
Source: Sports (Basel). 2025 Dec 8;13(12):441. doi: 10.3390/sports13120441 (PMC12737241; doi:10.3390/sports13120441)

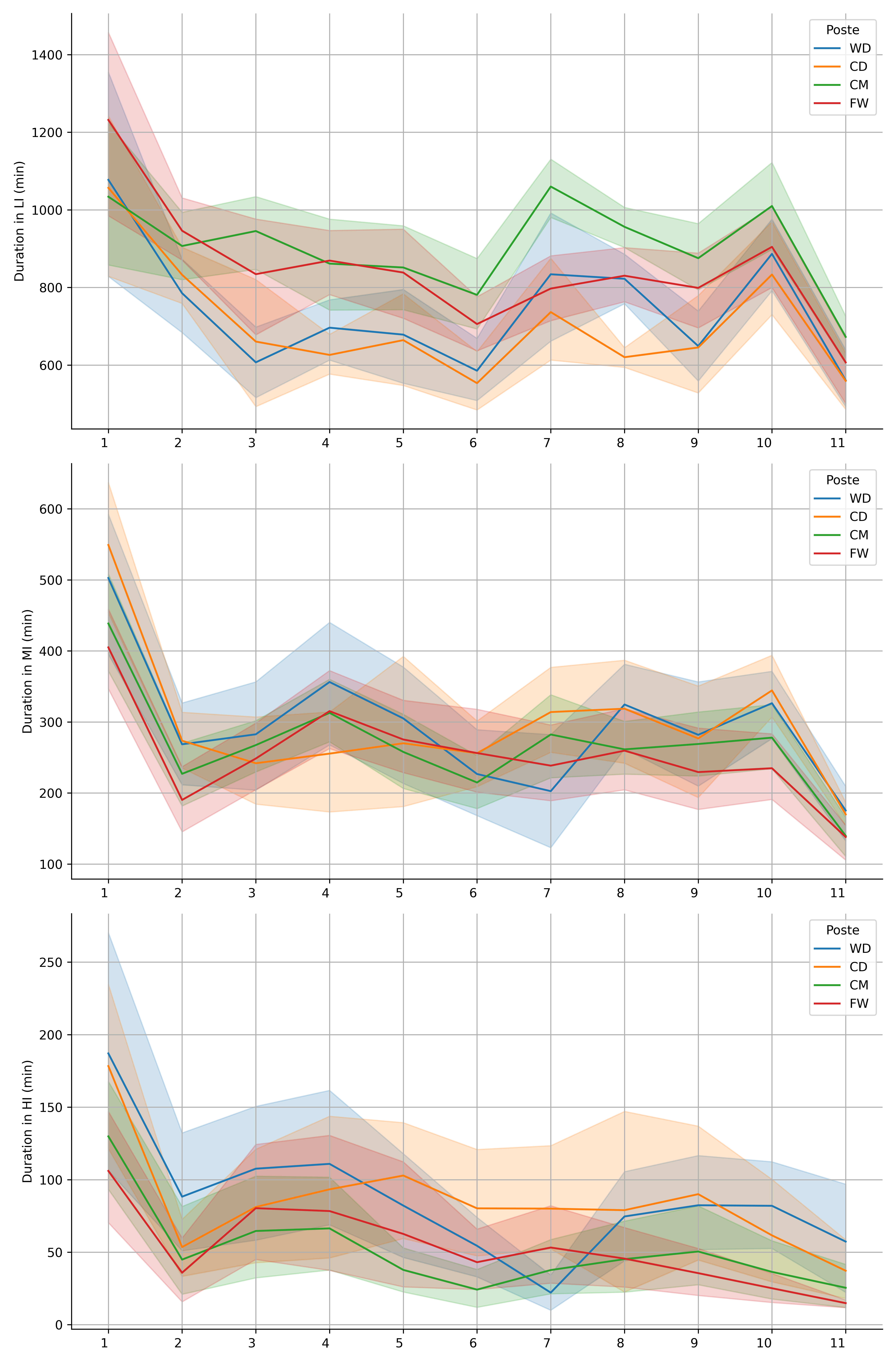

Supplement: Supplementary file 1 [file sports-13-00441-s001.zip › Supplementary Figure 1.png]

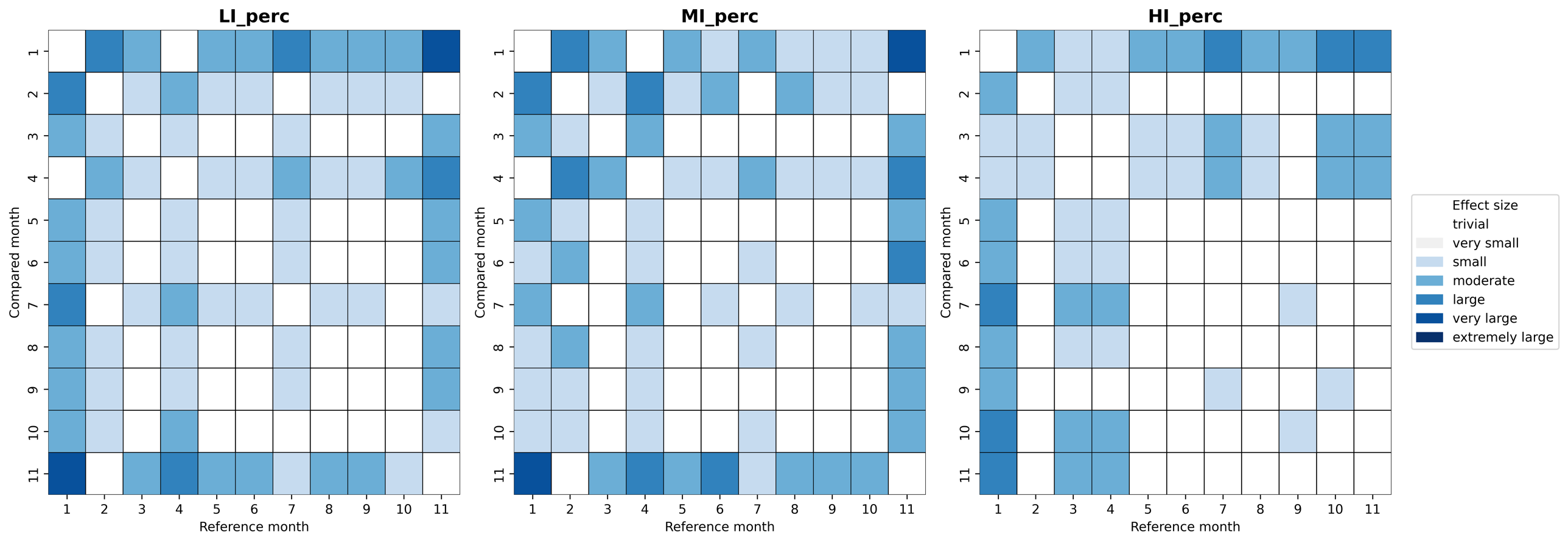

Supplement: Supplementary file 1 [file sports-13-00441-s001.zip › Supplementary Figure 2.png]

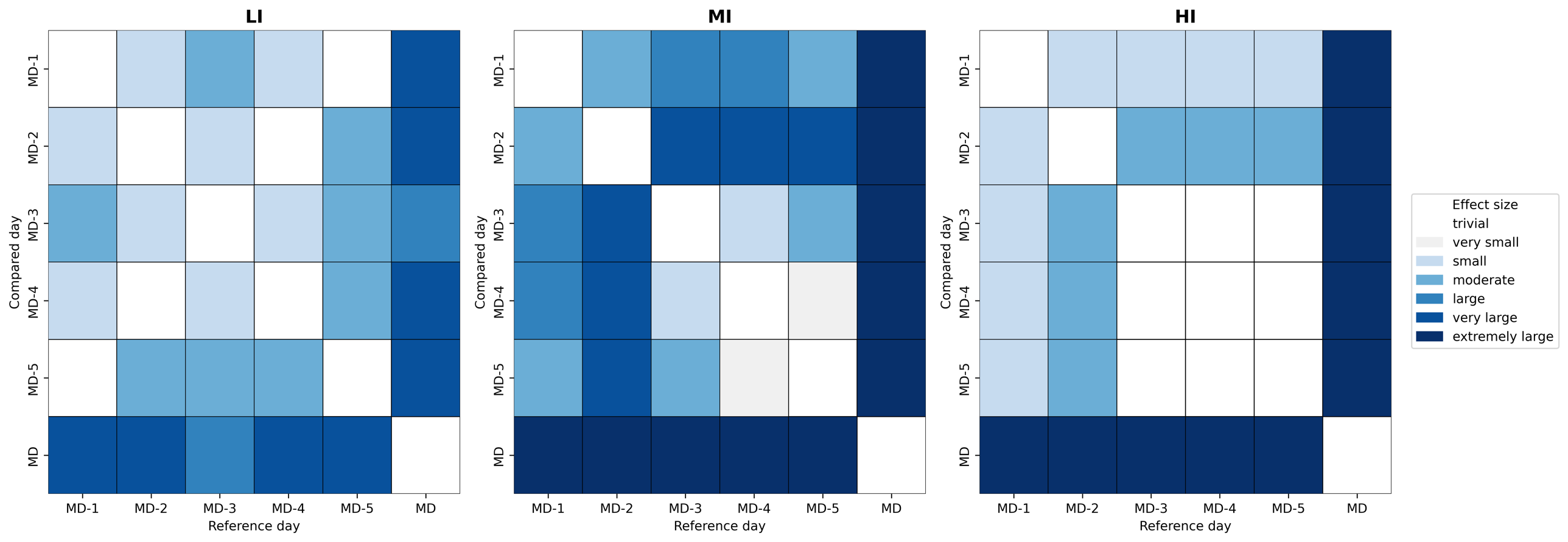

Supplement: Supplementary file 1 [file sports-13-00441-s001.zip › Supplementary Figure 3.png]

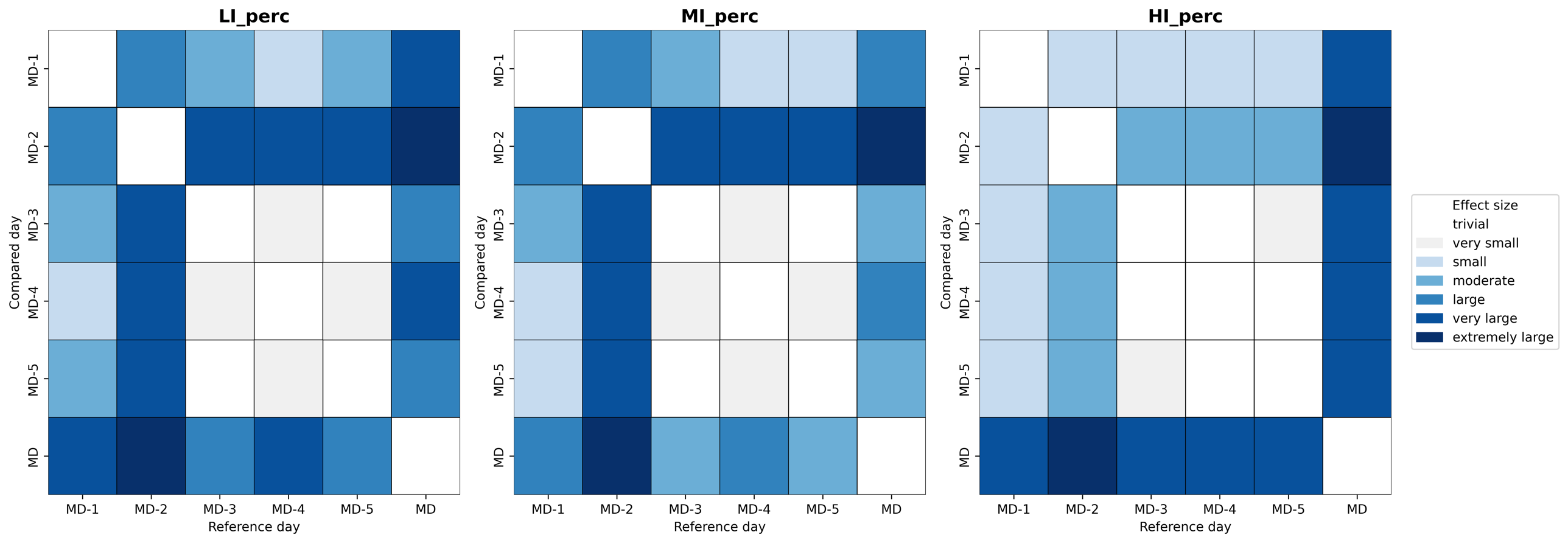

Supplement: Supplementary file 1 [file sports-13-00441-s001.zip › Supplementary Figure 4.png]

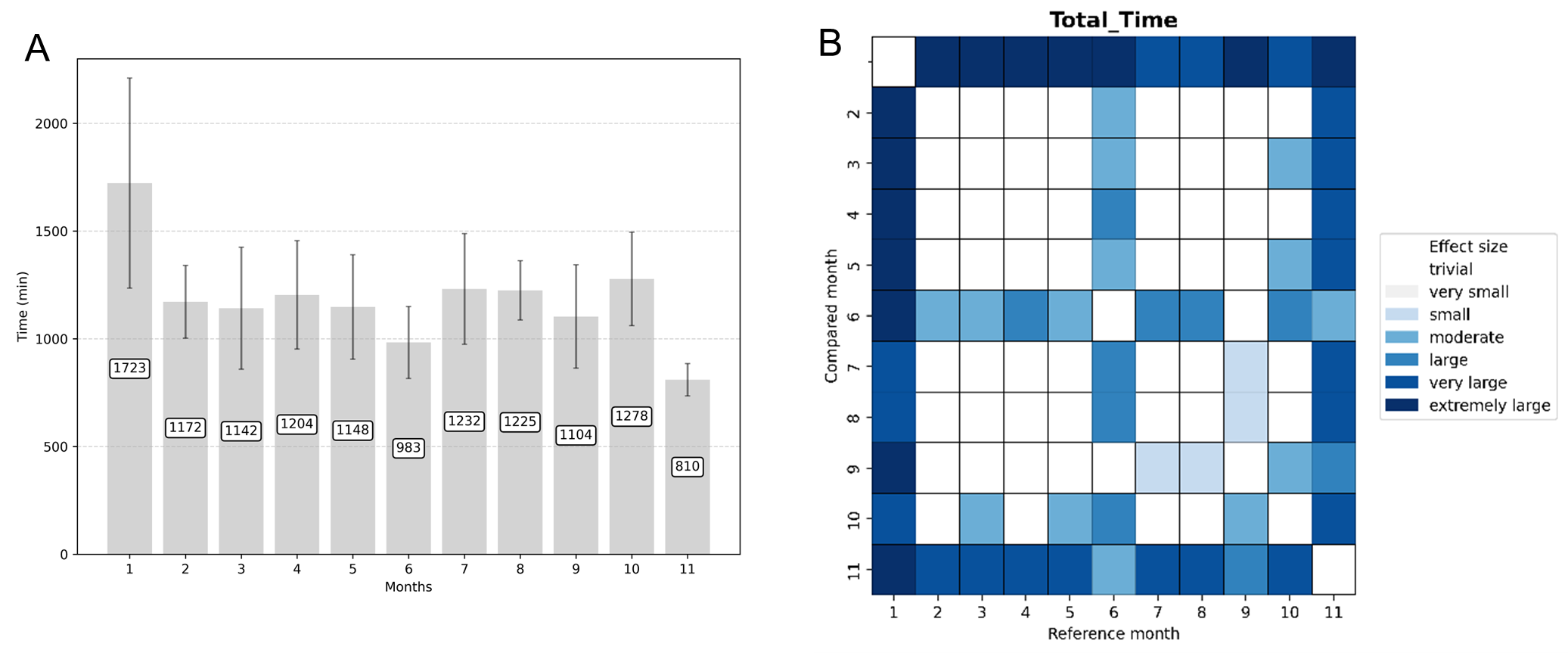

Supplement: Supplementary file 1 [file sports-13-00441-s001.zip › Supplementary Figure 5.png]
